# Dominant negative connexin26 mutation R75W causing severe hearing loss influences normal programmed cell death in postnatal organ of Corti

**DOI:** 10.1186/1471-2156-15-1

**Published:** 2014-01-03

**Authors:** Ayako Inoshita, Keiko Karasawa, Megumi Funakubo, Asuka Miwa, Katsuhisa Ikeda, Kazusaku Kamiya

**Affiliations:** 1 Department of Otorhinolaryngology, Juntendo University Faculty of Medicine, Hongo 2-1-1, Bunkyo-ku, Tokyo 113-8431, Japan

**Keywords:** Apoptosis, Hereditary hearing loss, Gjb2, Greater epithelial ridge, Mouse, Organ of corti

## Abstract

**Background:**

The greater epithelial ridge (GER) is a developmental structure in the maturation of the organ of Corti. Situated near the inner hair cells of neonatal mice, the GER undergoes a wave of apoptosis after postnatal day 8 (P8). We evaluated the GER from P8 to P12 in transgenic mice that carry the R75W + mutation, a dominant-negative mutation of human *gap junction protein, beta 2, 26 kDa* (*GJB2*) (also known as *connexin 26* or *CX26*). Cx26 facilitate intercellular communication within the mammalian auditory organ.

**Results:**

In both non-transgenic (non-Tg) and R75W + mice, some GER cells exhibited apoptotic characteristics at P8. In the GER of non-Tg mice, both the total number of cells and the number of apoptotic cells decreased from P8 to P12. In contrast, apoptotic cells were still clearly evident in the GER of R75W + mice at P12. In R75W + mice, therefore, apoptosis in the GER persisted until a later stage of cochlear development. In addition, the GER of R75W + mice exhibited morphological signs of retention, which may have resulted from diminished levels of apoptosis and/or promotion of cell proliferation during embryogenesis and early postnatal stages of development.

**Conclusions:**

Here we demonstrate that Cx26 dysfunction is associated with delayed apoptosis of GER cells and GER retention. This is the first demonstration that Cx26 may regulate cell proliferation and apoptosis during development of the cochlea.

## Background

Hereditary deafness affects about 1 in 2,000 children, and mutations in the *gap junction protein, beta 2, 26 kDa* gene (*GJB2*), also known as *connexin 26* (*CX26*), are the most common genetic causes of congenital bilateral non-syndromic sensorineural hearing loss. Gap junctions play important roles during the maturation and differentiation of developing tissues [1-5]. In the mammalian cochlea, we have demonstrated that a dominant-negative *GJB2* mutation results in incomplete postnatal development of the cochlear organ of Corti by R75W + transgenic mice, which carry a dominant-negative mutation of human *CX26*[6,7]. The organ of Corti in R75W + mice is reduced in height and has an increased midmodiolar-sectional area. In addition, several cochlear structures are absent in R75W + mice (including the tunnel of Corti, Nuel’s space, and spaces surrounding the outer hair cells), and the number of microtubules within the inner pillar cells is significantly reduced. Morphometric changes in R75W + mice likely result from collapse of the organ of Corti and enlargement of support cells causing impaired distortion product otoacoustic emission [8], although the underlying mechanisms remain unclear.

The greater epithelial ridge (GER) is a developmental structure that is important during maturation of the organ of Corti in mice. Present at birth, the GER typically disappears by postnatal day 10 (P10) [9,10]. In the mouse, most apoptosis in the developing cochlear system occurs during embryogenesis [11]. In the developing spiral ganglion cells of rats, however, 22% of programmed cell death was measured between P5 and P6 [12]. We have shown that development of normal hearing function in C3H/HeJ mice requires a wave of programmed cell death in the GER from P7 to P12. We also observed mitosis in the GER after P7, indicating that GER cells are both degenerating and regenerating until their eventual elimination at P12 [13].

Here, we evaluated postnatal changes in the GER of *Gjb2* mutant mice. We report that a dominant-negative *Gjb2* mutation induced GER retention, indicating that *Gjb2*-dependant apoptosis is critical for formation of a functionally and morphologically normal auditory system.

## Results

### Programmed cell death with Caspase-3 activation in GER

**Figure 1:**
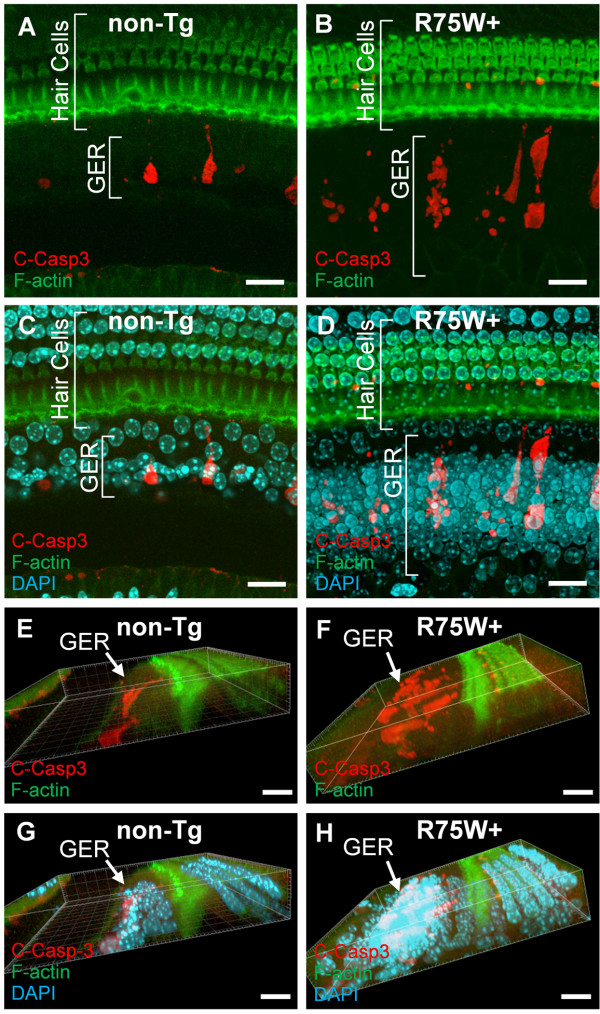
**Difference in programmed cell death with the activation of Caspase-3 in the GER of R75W + mouse at P11.** At P11, whole mount cochlear tissue of R75W + mice and the littermate controls was immunolabeled by anti-Cleaved-Caspase-3 (red, C-Casp3) with Phalloidin for F-actin (green) and DAPI for the nucleus (light green). Fifty slices of *z*-stack confocal images for the organ of Corti were collected at 0.5 μm intervals (total 25.5 μm depth from the surface of the organ of Corti). **(A-D)** The single image stacks for R75W + mouse **(B, D)** and the littermate controls **(A, C)** were constructed with LSM Image Browser (Zeiss). C-Casp3 and F-actin labelings are shown with **(C, D)** or without **(A, B)** nuclear labeling. **(E-H)** Three-dimensional images were constructed with the above confocal images of C-Casp3 and F-actin labeling with **(G, H)** or without **(E, F)** nuclear labeling. Bars indicate 20 μm.

To investigate whether the programmed cell death in GER of R75W + mice was affected, we performed whole mount immunostaining of the cochlea with anti-Cleaved Caspase-3 (C-Casp3) antibody. The z-stack confocal images and the three-dimensional images at P11 clearly showed the drastic change in the number of GER cells (Figure 1C,D), C-Casp3-positive cells (Figure 1A, B) and the shape of GER (Figure 1E, F). In R75W + mice, the number of GER cells and the C-Casp3 positive cells were higher than in the littermate control (Figure 1A-D) and the shape of the GER displayed an immature form which was flat and thick compare with the non-Tg littermate control, which was almost same form as the adult organ of Corti with a small ridge at the neural side of the hair cells (Figure 1E-H).

### Histological analysis of the GER

Apical turns of the cochleae were observed with cross sections. Histological examinations of H-E-stained cochlear sections revealed no gross differences in GER morphology between R75W + mice and non-transgenic (non-Tg) controls at P8 (Figure 1A,B). In both R75W + and non-Tg mice, a number of cells within the GER exhibited apoptotic characteristics (e.g., chromatin condensation) at P8 (Figure 1A,B, arrows). At P12, GER cells were clearly present in non-Tg mice, but both the number of cells in the GER and the area of the GER were visibly reduced compared to P8 (Figure 1C), and no apoptotic cells were detected. In contrast, there was less reduction in the GER size and apoptotic cells were visible in R75W + mice at P12 (Figure 1D, arrow).

TEM studies also revealed differences between non-Tg and R75W + mice at P12 (Figure 1E,F). Nuclei with highly condensed chromatin were detected in retained GER cells only in R75W + mice (Figure 1F, arrow). TEM images also indicated that the GER of R75W + mice at P12 was larger, both in cell number and in total area, than that of non-Tg mice (Figure 1E,F region within dotted line).

### Area of the GER

**Figure 2:**
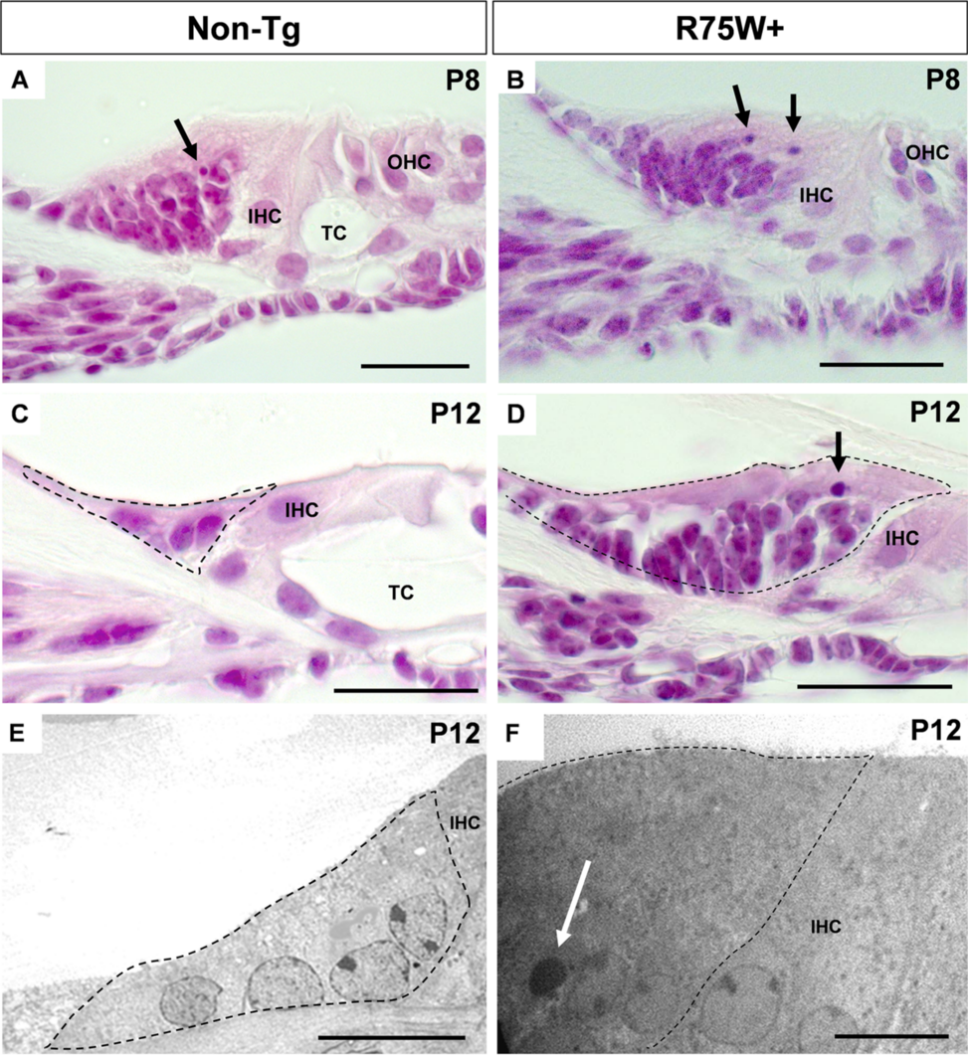
**Morphological analysis of the GER in R75W + transgenic mice. (A-D)** Hematoxylin and eosin staining of cochlear sections from R75W + and non-transgenic (non-Tg) mice. Apical turns in the cochlear were observed using microscopy. At P8, a fraction of the GER cells showed apoptotic characteristics (e.g., chromatin condensation) in both non-Tg and R75W + mice (**A**, **B**, arrows). IHC and OHC were detected in both non-Tg and R75W + mice **(A, B)**. TC was detected in non-Tg **(A)**, but not in R75W + **(B)**. At P12, the GER of non-Tg mice had 5–9 cells per section and no apoptosis **(C)**. In contrast, the GER (dotted line in D) of R75W + mice had 12–24 cells per section and had condensed nuclei indicative of apoptosis (**D**, arrow). TC is widely opened in non-Tg **(C)**, but is not detected in R75W + **(D). (E, F)** TEM images at P12 revealed no degenerative changes in non-Tg mice **(E)**, but nuclei with highly condensed chromatin were present in the enlarged GER of R75W + mice (**F**, arrow). The dotted line for C-E indicates the area of the GER. IHC, inner hair cell; OHC, outer hair cell; TC, tunnel of Corti. Scale bars = 25 μm **(A–D)** or 10 μm **(E, F)**.

**Figure 3:**
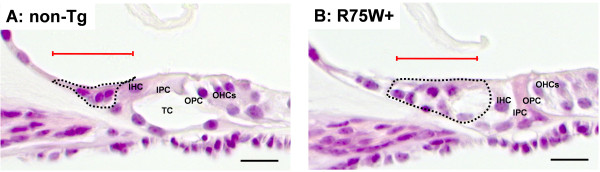
**Illustration of GER region used for measurement of area and cell number in non-Tg (A) and R75W + (B) mice at P12. **Apical turn in the cochlear were observed using microscopy. The dotted line indicates the area of the GER, and the red line indicates the 50-μm-long region in which cells were counted. IPC, inner pillar cell; OPC, outer pillar cell; IHC, inner hair cell; OHC, outer hair cell; TC, tunnel of Corti. Scale bars = 50 μm.

Total area of the GER was measured in non-Tg and R75W + mice at P8, P10, and P12 as illustrated in Figure 2 (see dotted area). When comparisons between time points were performed for either non-Tg mice or R75W + mice, no significant differences in GER size were detected (Figure 3). In comparisons between non-Tg and R75W + mice at P8 and P10, GER areas tended to be larger in R75W + mice, although these differences were not significant (Figure 3). At P12, however, the area of the GER was significantly larger in R75W + mice than in non-Tg mice (*p* = 0.043).

### Number of cells in the GER

**Figure 4:**
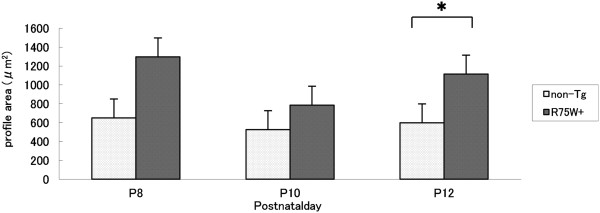
**Area of the GER as a function of age.** We examined GER areas in non-Tg and R75W + mice at P8 (n = 5), P10 (n = 5), and P12 (n = 5). For both non-Tg and R75W + mice, there were no significant differences among P8, P10, and P12. Between non-Tg and R75W + mice, the GER was significantly larger in R75W + mice at P12 (*p ≤ *0.05*). These data are expressed as mean ± SEM.

The total number of cells in the GER was counted for both non-Tg and R75W + mice at P8, P10, and P12 as illustrated in Figure 2 (see red line). When comparisons were made between non-Tg and R75W + mice at the three time points, no significant differences in cell number were detected at P8 or P10 (Figure 4). At P12, however, the GER of non-Tg mice had significantly fewer cells than the GER of R75W + mice (*p* = 0.050, Figure 4). The GER of non-Tg mice had 5–9 cells per section, whereas the GER of R75W + mice retained 12–24 cells per section (Figure 4).

### Apoptotic cells in the GER

**Figure 5:**
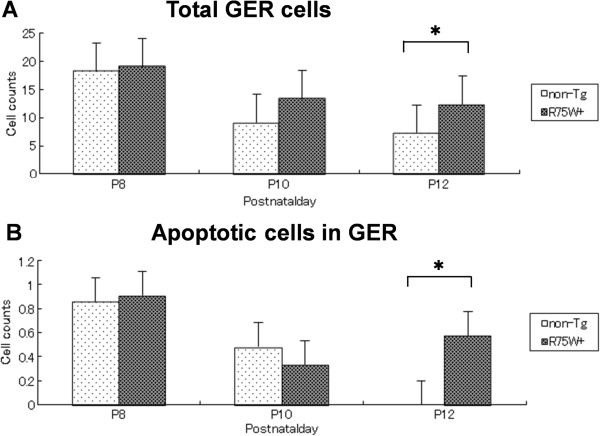
**Changes in number of GER cells and apoptotic cells. (A)** Number of cells in the GER as a function of age. We counted cells in the GER of both non-Tg and R75W + mice at P8 (n = 5), P10 (n = 5), and P12 (n = 5). For both non-Tg and R75W + mice, there were significantly fewer cells in the GER at P12 than at P8. At P12, there were significantly fewer cells in the GER of non-Tg mice than in R75W + mice. **(B)** Number of apoptotic cells in the GER as a function of age. We counted apoptotic cells in the GER of both non-Tg and R75W + mice at P8 (n = 5), P10 (n = 5), and P12 (n = 5). In non-Tg mice, there was significantly less apoptosis at P12 than at P8. In R75W + mice, however, there were no significant differences between P8, P10, and P12. At P12, there was significantly less apoptosis in the GER of non-Tg mice than in R75W + mice. These data are expressed as mean ± SEM. *denotes *p* ≤ *0.05*.

At P8, a small number of GER cells exhibited apoptotic characteristics (e.g., chromatin condensation) in both non-Tg and R75W + mice. In non-Tg mice the number of apoptotic cells declined after P8, with almost no apoptosis detected at P12 (Figure 5). In contrast, there were no significant differences in apoptotic nuclei in R75W + mice, and apoptotic nuclei were still detected in at the P12 time point. When the number of apoptotic cells in the GER was compared between non-Tg and R75W + mice, a significant difference was detected at P12 (*p* = 0.014; Figure 5).

## Discussion

In this study, we found elevated levels of programmed cell death in the GER of R75W + mice at P12 and the retention of GER at the same time.

The loss or impairment of gap junction-mediated intercellular communication has been associated with tumor progression in a number of contexts. Reduced expression of gap junction genes has been demonstrated in several human cancers, including gastric cancer (*CX32*) [14], prostatic adenocarcinoma (*CX43*) [15], brain glioma (*CX43*) [16], breast cancer (*CX43*) [17], and lung cancer (*CX32* and *CX43*) [18]. When these gene functions are restored, cell growth slows and more normal phenotypes arise [19,20]. Thus, *connexins* are generally considered tumor suppressor genes [21]. Transfection of *CX26* into a tumor cell lines typically results in growth suppression [22-24].

**Figure 6:**
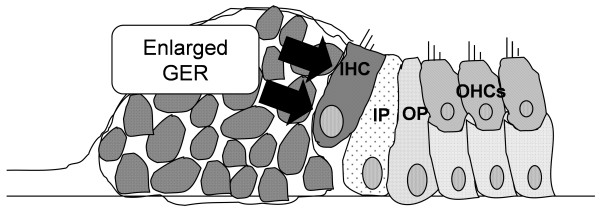
**Schematic diagram of the organ of Corti in R75W + mice.** IHC, inner hair cell; OHC, outer hair cell;IP, Inner Pillar cell, OP; Outer pillar cell.

Connexins have also been associated with programmed cell deatz [25,26]. In colorectal cancer cells, positive associations have been identified between *CX26* and the pro-apoptotic gene *BCL2-associated X protein* (*BAX*) and between *CX26* and the anti-apoptotic gene *BCL2-like 1* (*BCL2L1*) [27]. Wild-type gap junctions help regulate cellular growth, differentiation, tissue development, and apoptosis [28]. Finally, aberrant DNA methylation of the *CX26* promoter region, which leads to its inactivation, is involved in human cancers [29,30]. In this study, we demonstrate that a dominant-negative mutation of *Gjb2* induced retention in the mouse GER during the early postnatal stages of cochlear development. We propose the existence of an underlying mechanism to explain the disruption of the cyto-architecture in the organ of Corti in prelingual deafness caused by the *Gjb2* mutation (Figure 6). This clearly disrupted the cyto-architecture of the organ of Corti (Figure 1D, 2B). In wild-type mice, the height of the organ of Corti increases as the organ develops [31]. In R75W + mice, however, the height of the organ remained unchanged as development progressed, presumably because the tunnel of Corti collapsed [6]. In this regard, the cochleae of R75W + mice were quite similar to those of *caspase 3* (−/−) mice [32], in which the space that forms the tunnel of Corti is known to collapse, reducing the height of the organ of Corti. We have previously shown GER retention in mice that lack *caspase 3*[32]. The enlarged GER that characterizes *caspase 3* (−/−) mice may result from diminished apoptosis and/or elevated levels of cell proliferation during embryogenesis. Most apoptosis within the developing cochlear system of the mouse occurs during embryogenesis [11]. We have demonstrated, however, that apoptosis of GER cells during postnatal stages is essential for normal development of the cochlea [13,32]. The present study suggests that *Gjb2* dysfunction may promotes survival of GER cells by delaying apoptosis in the organ of Corti during postnatal stages of development.

It is still unclear why disruption of *Gjb2* results in hearing loss, particularly when other cochlear connexins (e.g., Cx30) can compensate for Cx26 dysfunction. One possibility is that Cx26 may be more involved in regulating cell proliferation and apoptosis in the organ of Corti than other connexins. Cx26 disruption, therefore, may delay apoptosis in the GER, collapse the tunnel of Corti, and ultimately disrupt the cyto-architecture of the organ of Corti. Additional experiments are required to further elucidate the mechanisms by which the inner ear develops.

## Conclusions

The present findings strongly suggest that the dominant-negative R75W + mutation of *Cx26* delays programmed cell death around the organ of Corti. This results in collapse of the organ of Corti. These findings indicate that CX26 may play a critical role in regulating cell proliferation and apoptosis during cochlear development.

## Methods

### Animals

All mice were obtained from a breeding colony of R75W + mice which were heterozygous [7] and maintained at the Institute for Animal Reproduction (Ibaraki, Japan). R75W + mice were maintained in a mixed C57BL/6 background and intercrossed to generate R75W + animals. Littermates of wild type mice were used as controls.

The animals were genotyped using DNA obtained from tail clips and amplified using a Tissue PCR Kit (Sigma, Saint Louis, MO, USA) as described [7]. All experimental protocols were approved by the Institutional Animal Care and Use Committee at Juntendo University, and were conducted in accordance with the US National Institutes of Health Guidelines for the Care and Use of Laboratory Animals. All reasonable efforts were taken to minimize the number of animals used, as well as their suffering. Five mice were examined in each age group.

### Immunohistochemistry

Mice were anesthetized, killed and inner-ear tissues were removed. The cochleae were further dissected and fixed in 4% paraformaldehyde”. Immunofluorescence staining with antibody against cleaved Caspase-3 (C-Casp3, rabbit IgG, Promega) was performed on whole-mount preparations of the finely dissected organ of Corti. We incubated the tissues in the antibody solutions for 1 h after blocking. As secondary antibodies, we used Cy3–conjugated anti rabbit IgG (Sigma Aldrich). F-actin was visualized with Alexa 633–conjugated Phalloidin (Life technologies). Fluorescence confocal images were obtained with a LSM510-META confocal microscope (Carl Zeiss). Some of the green fluorescence in Cx26R75W + mice indicated the pseudocolor obtained from the signal of Alexa 633–conjugated secondary antibodies (Invitrogen), because these mice have ubiquitous EGFP expression from their transgene. z-stacks of images were collected at 0.5 μm intervals, and the single image stacks were constructed with LSM Image Browser (Zeiss); three-dimensional images and videos were constructed with IMARIS software (Bitplane). We analyzed at least six samples from three animals, and representative images are shown. The compared images were photographed and processed using identical parameters. Three-dimensional images were constructed with z-stacked confocal images by IMARIS (Bitplane).

### Light microscopy

Animals were deeply anesthetized with an intraperitoneal injection of ketamine (100 mg/kg) and xylazine (10 mg/kg). They were then perfused intracardially with 0.1 M phosphate-buffered saline (PBS, 137 mM NaCl, 2.7 mM KCl, 1.47 mM KH2PO4, 8.1 mM Na2HPO4, pH 7.2) followed by 4% paraformaldehyde (PFA) in 0.1 M phosphate buffer (PB, 0.2 M Na2HPO4, 0.2 M NaH2PO4, pH 7.4). The mice were decapitated and their cochleae dissected under a microscope. Dissected cochleae were fixed in 4% PFA at room temperature overnight, decalcified in 0.12 M ethylenediaminetetraacetic acid in PBS (pH 7.0) for 1 week, dehydrated in a graded ethanol series, and finally embedded in paraffin. Serial sections (6 μm) were stained with hematoxylin and eosin (H-E).

### Transmission electron microscopy (TEM)

Animals were deeply anesthetized and perfused intracardially with PBS followed by 4% PFA and 2% glutaraldehyde (GA) in PB. Cochleae were opened, flushed with buffered 4% PFA 2% GA, and fixed an additional 2 h at room temperature. After washing, specimens were post-fixed in 2% OsO4 in PB for 1.5 h, then dehydrated in a graded ethanol series and embedded in Epon. Serial sections (1 μm) were stained using uranyl acetate and lead citrate, and examined by electron microscopy (H-7100, Hitachi, Tokyo, Japan).

### Quantification and statistical analysis

We measured the area, the number of cells, and the number of apoptotic cells in the GER of both mutant and wild-type littermates. Measurements were performed at P8, P10, and P12. All counts were performed using mid-modiolar sections of H-E-stained cochleae.

To measure GER area, digital light micrograph images of the organ of Corti were captured using NIS Elements-D software (Nicon, Tokyo, Japan). Cell counts were obtained by analyzing a 50-μm segment of the GER extending from the inner hair cell toward the modiolus using a 100× objective. The middle turns of the cochlea were analyzed. Results are expressed as mean ± SEM. Statistical significance between two groups was analyzed using the Mann–Whitney *U* test. Analyses between three groups were performed using the Kruskal-Wallis test followed by Dunn’s Multiple Comparison test. A *p* value ≤0.05 was considered significant. Three animals from P8 and P10, and four animals from P12, were analyzed. Five sections were analyzed for each mouse.

## Abbreviations

GER: Greater epithelial ridge; Cx26: Connexin26; EDTA: Ethylenediaminetetraacetic acid; GA: Glutaraldehyde; GJB2: Connexin26 gene; H-E: Hematoxylin and eosin; P: Postnatal; PB: Phosphate buffer; PBS: Phosphate-buffered saline; PFA: Paraformaldehyde; TEM: Transmission electron microscopy; Tg: Transgenic.

## Competing interests

The authors declare that they have no competing financial interests.

## Authors’ contributions

AI carried out complement functional activity, quantitative analysis, all statistical analysis and wrote the manuscript. KK secondary principal investigator performed the immunohistochemistry and image processing and reviewed the manuscript. KK, MF, and AM performed animal experiments and immunohistochemistry. KI primary principal investigator advised on the study. All authors read and approved the manuscript.
